# Epigenetic age acceleration and metabolic syndrome in the coronary artery risk development in young adults study

**DOI:** 10.1186/s13148-019-0767-1

**Published:** 2019-11-15

**Authors:** Drew R. Nannini, Brian T. Joyce, Yinan Zheng, Tao Gao, Lei Liu, Grace Yoon, Tianxiao Huan, Jiantao Ma, David R. Jacobs, John T. Wilkins, Jim Ren, Kai Zhang, Sadiya S. Khan, Norrina Bai Allen, Steve Horvath, Donald M. Lloyd-Jones, Philip Greenland, Lifang Hou

**Affiliations:** 1 Department of Preventive Medicine and Robert H. Lurie Comprehensive Cancer Center, Northwestern University Feinberg School of Medicine, 680 N. Lake Shore Dr., Suite 1400, Chicago, IL 60611 USA; 2 Division of Biostatistics, Washington University, 660 S. Euclid Ave, St. Louis, MO 63110 USA; 3 Department of Statistics, Texas A&M University, 3143 TAMU, College Station, TX 77843 USA; 4 The National Heart, Lung, and Blood Institute’s Framingham Heart Study and Population Sciences Branch, Framingham, MA USA; 5 Division of Epidemiology and Community Health, School of Public Health, University of Minnesota, 1300 S 2nd St, Minneapolis, MN 55454 USA; 6 Department of Preventive Medicine, Northwestern University Feinberg School of Medicine, 680 N. Lake Shore Dr., Suite 1400, Chicago, IL 60611 USA; 7 Department of Epidemiology, Human Genetics and Environmental Sciences, School of Public Health, The University of Texas Health Science Center at Houston, 1200 Pressler Street, RAS W606, Houston, TX 77030 USA; 8 Department of Human Genetics, David Geffen School of Medicine, University of California Los Angeles, Los Angeles, CA 90095 USA; 9 Department of Biostatistics, Fielding School of Public Health, University of California Los Angeles, Los Angeles, CA 90095 USA

**Keywords:** Epigenetic age acceleration, Metabolic syndrome, CARDIA, DNA methylation

## Abstract

**Background:**

The metabolic syndrome (MetS) is a collection of metabolic disturbances that can lead to various cardiovascular diseases. Previous studies have shown a more adverse metabolic risk profile is associated with more advanced biological aging. The associations between epigenetic biomarkers of age with MetS, however, are not well understood. We therefore investigated the associations between epigenetic age acceleration and MetS severity score and incident MetS.

**Results:**

A subset of study participants with available whole blood at examination years 15 and 20 from the Coronary Artery Risk Development in Young Adults Study underwent epigenomic profiling using the Illumina MethylationEPIC Beadchip (~ 850,000 sites). Intrinsic and extrinsic epigenetic age acceleration (IEAA and EEAA) were calculated from DNA methylation levels. The MetS severity score was positively associated with IEAA at years 15 (*P* = 0.016) and 20 (*P* = 0.016) and EEAA at year 20 (*P* = 0.040) in cross-sectional analysis. IEAA at year 20 was significantly associated with incident MetS at year 30 (OR = 1.05 [95% CI 1.01, 1.10], *P* = 0.028).

**Conclusions:**

To our knowledge, this is the first report of the longitudinal association between epigenetic age acceleration and MetS. These findings suggest that a higher MetS severity score is associated with accelerated epigenetic aging and such aging may play a role in the development of metabolic disorders, potentially serving as a useful biomarker of and early detection tool for future MetS.

## Background

The metabolic syndrome (MetS) is defined as a collection of metabolic disturbances that includes hypertension, elevated plasma glucose, dyslipidemia, and abdominal obesity [1]. Individuals with MetS are at higher risk for type 2 diabetes, several types of cancer, and cardiovascular morbidity and mortality [2–6]. The prevalence of MetS has increased over the past several decades worldwide and currently affects more than one third of all adults and nearly half of those 60 years of age and older in the USA [7–9]. Given the number of individuals affected by this condition and the aging population, understanding the underlying molecular determinants and biological processes of MetS may lead to the development of novel markers of risk and prevention tools for this condition.

Substantial interindividual differences in age-associated metabolic dysregulation exist in older adults, which often exceeds the variation in younger adults, suggesting that chronological age may not be an adequate index of physiologic aging. Previous studies have investigated the association between leukocyte telomere length, a measure of biological age, and the components of MetS. For example, shorter leukocyte telomere length was found to be associated with higher triglycerides and fasting glucose, lower high-density lipoprotein (HDL) cholesterol, and obesity, as well as the total number of MetS components and presence of MetS [10–14]. These findings suggest that a more adverse metabolic risk profile is associated with accelerated biological aging. Assessment of other measures of aging may further our understanding of the association between the aging process and MetS.

Research into epigenetic mechanisms has led to the identification of age-related DNA methylation profiles/signatures that correlate with chronological age [15]. Specifically, the weighted average of DNA methylation levels at multiple CpG sites is commonly used to calculate epigenetic age as well as the discrepancy between this biological age measure and chronological age, referred to as epigenetic age acceleration (EAA). Intrinsic epigenetic age acceleration (IEAA) and extrinsic epigenetic age acceleration (EEAA), two measures of EAA, have been widely used to investigate the associations between epigenetic aging and numerous traits. IEAA has been proposed to capture cell-intrinsic properties of the aging process, while EEAA reflects immune system aging. Overall, slower epigenetic age acceleration (i.e., negative values) has been associated with lifestyle factors such as diet, alcohol consumption, physical activity, and educational attainment [16], whereas faster epigenetic age acceleration (i.e., positive values) has been associated with multiple age-related health outcomes including frailty, cancer, lung function, cognition, and all-cause mortality [17–20]. While accelerated epigenetic aging has been associated with lifestyle factors and diseases related to MetS, the relationship between EAA and this metabolic condition is currently not clearly delineated.

In this study, we investigated the cross-sectional and longitudinal associations between epigenetic age acceleration and MetS by leveraging blood epigenomewide DNA methylation data from the Coronary Artery Risk Development in Young Adults (CARDIA) study. We hypothesized that the number of MetS components (MetS severity score) is positively associated with epigenetic age acceleration and that individuals with accelerated epigenetic age are more likely to develop incident MetS.

## Results

### Sample characteristics

Summary characteristics for the study participants who underwent methylation profiling and those who did not at examination years 15 and 20 from the CARDIA cohort are presented in Table 1. Overall, 1042 and 957 participants who underwent methylation profiling had data quality acceptable for further analysis at years 15 and 20, respectively. Year 15 participants with methylation data had a mean (standard deviation) age of 40.4 (3.5) years, were 51.3% female, had 15.1 (2.5) years of education, were 59.3% white, and 62.8% had never smoked; similar characteristics were found for year 20 participants with available methylation data. At year 15, 35% of participants with methylation data had two or more MetS components, compared to 46% of participants at year 20. Compared to those without methylation data at year 15, those with methylation data were older and had lower proportions of female and black participants, more years of education, lower systolic blood pressure, and higher triglycerides (*P* < 0.05). Similarly, the subset with methylation data had lower proportions of female and black participants, higher total cholesterol and triglycerides, and lower HDL compared to those without methylation data at year 20 (*P* < 0.05). Additional file 1: Table S1 presents the summary characteristics after adjusting for sex and race. Overall, only total cholesterol and triglycerides differed between methylation groups at year 20 after adjustment for sex and race. We found no significant differences in the remaining sample characteristics, i.e., center, smoking status, alcohol, physical activity, body mass index, glucose, waist circumference, or MetS, at either year 15 or 20. Additional file 2: Figure S1 presents a scatterplot matrix displaying the relationships between chronological age, epigenetic age as calculated by Horvath’s and Hannum’s method, and the MetS severity score at examination years 15 and 20.

**Table 1:** Comparison of study sample characteristics between participants who underwent methylation profiling and participants who did not at examination years 15 and 20

Characteristic^a^	Year 15 cohort			Year 20 cohort		
	Without methylation	With methylation	*P*	Without methylation	With methylation	*P*
*N*	2630	1042		2592	957	
Female, *n* (%)	1517 (57.7)	535 (51.3)	0.0005	1524 (58.8)	490 (51.2)	0.0001
Race, *n* (%)						
White	1324 (50.3)	618 (59.3)		1334 (51.5)	564 (58.9)	
Black	1306 (49.7)	424 (40.7)	0.0001	1258 (48.5)	393 (41.1)	0.0001
Age, mean (SD), years	40.1 (3.7)	40.4 (3.5)	0.04	45.1 (3.7)	45.4 (3.5)	0.06
Epigenetic age, mean (SD), years^b^	N/A	45.4 (5.5)		N/A	49.4 (6.1)	
IEAA, mean (SD), years	N/A	0 (4.6)		N/A	0 (5.4)	
EEAA, mean (SD), years	N/A	0 (6.4)		N/A	0 (5.4)	
Education, mean (SD), years	14.8 (2.5)	15.1 (2.5)	0.007	15.0 (2.6)	15.1 (2.5)	0.20
Center, *n* (%)						
Birmingham, AL	603 (22.9)	255 (24.5)		597 (23.0)	222 (23.2)	
Chicago, IL	591 (22.5)	225 (21.6)		585 (22.6)	208 (21.7)	
Minneapolis, MN	715 (27.2)	278 (26.7)		667 (25.7)	258 (27.0)	
Oakland, CA	721 (27.4)	284 (27.3)	0.78	743 (28.7)	269 (28.1)	0.87
Smoking status, *n* (%)						
Never	1540 (58.7)	653 (62.8)		1580 (61.5)	570 (60.2)	
Former	484 (18.4)	181 (17.4)		491 (19.1)	191 (20.2)	
Current	601 (22.9)	206 (19.8)	0.06	497 (19.4)	186 (19.6)	0.73
Alcohol, mean (SD), mL/day	10.5 (25.7)	12.1 (22.6)	0.08	10.0 (18.2)	11.1 (18.4)	0.10
Physical activity, mean (SD), total intensity score	346.3 (287.1)	350.0 (274.7)	0.72	331.2 (273.3)	348.6 (276.1)	0.09
BMI, mean (SD), kg/m^2^	28.8 (7.1)	28.5 (6.2)	0.20	29.5 (7.5)	29.3 (6.5)	0.32
SBP, mean (SD), mmHg	113.6 (15.4)	112.3 (13.6)	0.001	116.8 (15.4)	116.4 (14.9)	0.53
DBP, mean (SD), mmHg	74.7 (11.9)	74.1 (10.8)	0.14	73.3 (11.6)	72.7 (11.2)	0.17
Total cholesterol, mean (SD), mg/dL	184.0 (35.9)	186.4 (35.5)	0.07	184.7 (34.6)	188.3 (36.1)	0.007
HDL cholesterol, mean (SD), mg/dL	50.9 (14.7)	50.1 (14.1)	0.10	54.6 (16.6)	53.2 (16.7)	0.03
Triglycerides, mean (SD), mg/dL	103.2 (92.5)	110.9 (93.4)	0.02	105.9 (72.6)	119.0 (95.4)	0.0001
Glucose, mean (SD), mg/dL	86.8 (21.9)	86.3 (18.4)	0.42	98.1 (27.4)	97.9 (23.8)	0.89
Waist circumference, mean (SD), cm	89.5 (16.0)	89.5 (13.9)	0.94	91.8 (15.9)	92.2 (14.6)	0.48
Metabolic syndrome						
Prevalence, *n* (%)	426 (16.8)	164 (15.9)	0.53	625 (24.7)	253 (26.5)	0.28
Severity score, median (IQR)	1 (0, 2)	1 (0, 2)		1 (0, 2)	1 (0, 3)	
0 components, *n* (%)	942 (37.0)	366 (35.5)		757 (30.0)	278 (29.1)	
1 component, *n* (%)	678 (26.7)	307 (29.8)		669 (26.4)	237 (24.8)	
2 components, *n* (%)	498 (19.6)	195 (18.9)		482 (19.0)	188 (19.7)	
3 components, *n* (%)	279 (11.0)	108 (10.5)		334 (13.2)	146 (15.3)	
4 components, *n* (%)	115 (4.5)	49 (4.8)		219 (8.7)	78 (8.2)	
5 components, *n* (%)	32 (1.3)	7 (0.7)	0.33	72 (2.8)	29 (3.0)	0.63

^a^Study sample characteristics were measured at years 15 and 20 of CARDIA, respectively

^b^DNA methylation age as predicted by Horvath’s method

### Metabolic syndrome severity score

Table 2 presents the results for the cross-sectional analyses of the MetS severity score and epigenetic age acceleration, as well as results from the generalized estimating equation (GEE) analyses using measurements at two time points. After adjusting for sex, race, center, education, smoking status, alcohol consumption, and physical activity, the MetS severity score was positively associated with IEAA but not EEAA at year 15 (*P* = 0.016 and *P* = 0.209, respectively). That is, each additional MetS component was associated with a 0.29 [95% CI 0.05, 0.53]-year gain in IEAA. The MetS severity score was also positively associated with both IEAA and EEAA at year 20 after adjusting for covariates, with a 0.27 [95% CI 0.05, 0.49]-year gain in IEAA (*P* = 0.016) and a 0.25 [95% CI 0.01, 0.49]-year gain in EEAA (*P* = 0.040) for each additional MetS component. Moreover, after adjusting for covariates, the MetS severity score was significantly and positively associated with both IEAA and EEAA using measurements at two time points. Specifically, there was a respective 0.29 [95% CI 0.11, 0.47]-year (*P* = 0.002) and a 0.21 [95% CI 0.02, 0.41]-year (*P* = 0.034) gain in IEAA and EEAA per each additional MetS component. Interaction analyses yielded no significant associations.

**Table 2:** Cross-sectional results for the association between EAA and the MetS severity score at examination years 15 and 20

	IEAA		EEAA	
	*β* [95% CI]	*P*	*β* [95% CI]	*P*
Year 15				
Crude model	0.31 [0.08, 0.53]	0.008	0.16 [− 0.11, 0.43]	0.236
Adjusted model^a^	0.29 [0.05, 0.53]	0.016	0.17 [− 0.10, 0.44]	0.209
Year 20				
Crude model	0.32 [0.11, 0.53]	0.003	0.24 [0.00, 0.47]	0.047
Adjusted model^a^	0.27 [0.05, 0.49]	0.016	0.25 [0.01, 0.49]	0.040
GEE				
Crude model	0.31 [0.14, 0.48]	< 0.001	0.21 [0.01, 0.41]	0.036
Adjusted model^a^	0.29 [0.11, 0.47]	0.002	0.21 [0.02, 0.41]	0.034

^a^Results are adjusted for sex, race, center, education, smoking status, alcohol consumption, and physical activity

Beta coefficients represent the amount of years gained per each additional MetS component

**Fig. 1:**
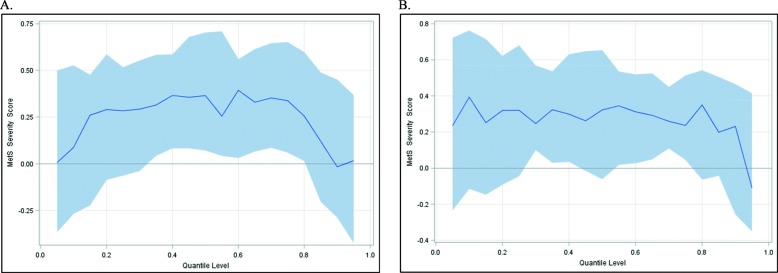
Estimated parameters by quantile with 95% confidence limits for the effect of the MetS severity score on IEAA. Quantile regression plots for the MetS severity score at years **a** 15 and **b** 20. The *x-*axis represents the quantile scale, and the *y*-axis represents the effect of the MetS severity score on IEAA for a given quantile. Results are adjusted for sex, race, center, education, smoking status, alcohol consumption, and physical activity

We performed quantile regression to further evaluate the effect of the MetS severity score on EAA. Figure 1 displays plots generated from the quantile regression analyses for the MetS severity score at years 15 and 20 for IEAA. We plotted regression estimates for 19 quantiles ranging from 0.05 to 0.95. As illustrated in the plots, the overall pattern depicts that the MetS severity score has a positive effect on IEAA at both years 15 and 20. For year 15, the effect of the MetS severity score on IEAA in the middle range of the distribution can be four times greater than the lower and upper tails of the distribution (0.36 vs 0.09 year gain, respectively). The effect of the MetS severity score at year 20 appears to be relatively flat, with a 0.27-year gain in IEAA for nearly all quantiles. Similar overall patterns were observed for the effect of the MetS severity score on EEAA at years 15 and 20 (Additional file 3: Figure S2.)

### Metabolic syndrome

We next evaluated whether epigenetic age acceleration at years 15 and 20 was associated with incident MetS status at years 25 and 30. Table 3 displays the association results for both the logistic and GEE approaches. Neither IEAA nor EEAA at year 15 was significantly associated with MetS status at years 25 and 30. Similarly, neither IEAA nor EEAA at year 20 was associated with MetS status at year 25; however, IEAA at year 20 was significantly associated with MetS status at year 30. That is, every 1-year gain in IEAA was associated with a 5% greater odds of MetS (OR = 1.05 [95% CI 1.01, 1.10]; *P* = 0.028). Neither IEAA nor EEAA was significantly associated with MetS status at year 25 using measurements at two time points. IEAA, however, was significantly associated with MetS status at year 30. Specifically, a 1-year gain in IEAA was associated with a 4% greater odds of MetS (OR = 1.04 [95% CI 1.01, 1.07]; *P* = 0.024). Sex and race interactions with IEAA and EEAA were non-significant.

**Table 3:** Prospective results for the association between MetS status at examination years 25 and 30 and EAA at years 15 and 20

	MetS_25_		MetS_30_	
	OR [95% CI]	*P*	OR [95% CI]	*P*
Year 15				
IEAA	1.01 [0.97, 1.05]	0.666	1.03 [0.99, 1.07]	0.111
EEAA	1.02 [0.98, 1.05]	0.374	1.01 [0.98, 1.04]	0.496
Year 20				
IEAA	1.04 [0.99, 1.09]	0.171	1.05 [1.01, 1.10]	0.028
EEAA	1.02 [0.97, 1.07]	0.538	1.03 [0.99, 1.07]	0.212
GEE				
IEAA	1.02 [0.98, 1.05]	0.300	1.04 [1.01, 1.07]	0.024
EEAA	1.02 [0.99, 1.04]	0.281	1.02 [0.99, 1.04]	0.255

Results are adjusted for sex, race, center, education, smoking status, alcohol consumption, and physical activity

## Discussion

Our results indicate associations between epigenetic age acceleration and MetS severity score and incident MetS, independent of known risk factors. We observed positive cross-sectional associations between MetS severity score and IEAA at year 15 and both measures of epigenetic age acceleration (IEAA and EEAA) at year 20, with a higher severity score associated with more advanced epigenetic age acceleration. We also observed a positive association between the MetS severity score and both IEAA and EEAA with repeated measures. Additionally, we found that IEAA was associated with incident MetS, with a positive association between IEAA and MetS status using GEE. These findings suggest associations between EAA and MetS both cross-sectionally and longitudinally.

The MetS severity score was positively associated with epigenetic age acceleration in our study, indicating greater accelerated epigenetic aging for each additional MetS component. Our finding that the MetS severity score is associated with biological aging is in agreement with previous findings [10]. Moreover, our results are consistent with a recent study that reported positive associations between epigenetic age acceleration and the number of MetS components [16]. Although it is generally accepted that MetS induces precocious aging, the exact mechanisms through which this occurs are unclear [21]. One theory suggests that excess reactive oxygen species may contribute to metabolic dysregulation, cell damage, and consequently aging [22]. Similarly, DNA methylation may partially mediate the effects that oxidative stress may have on the metabolic dysregulation found in MetS [23]. Together, the current and previous findings demonstrate that metabolic risk factors may have negative impacts on biological aging. Moreover, understanding the effect of metabolic risk factors on epigenetic modifications may lead to interventions that slow the aging process at the cellular and/or molecular levels and potentially prevent age-related diseases.

We further conducted quantile regression to investigate the association between MetS severity score and epigenetic age acceleration. In quantile regression, the conditional quantiles of an outcome are modeled, compared to ordinary least squares regression, which models the conditional mean of the response variable. As such, the former approach provides a more detailed picture of the conditional distribution of a response variable (EAA) for a given independent variable (MetS severity score) and is useful when the change in response varies by quantile. In the current analysis, there was an overall positive association of MetS severity score on epigenetic age acceleration at both years 15 and 20 with linear regression. This effect, however, varied across the MetS severity score-IEAA distribution at year 15 with the MetS severity score exhibiting a larger effect in the middle of the distribution compared to the tails. This suggests that an additional MetS component has a smaller effect among individuals with highly advanced and highly slowed epigenetic age acceleration but a greater impact among individuals with modest to no epigenetic age acceleration. This finding suggests that individuals with relatively limited accelerated epigenetic age may be most affected by metabolic risk factors with regard to epigenetic aging, a finding that is not captured using traditional linear regression. As such, lifestyle modifications that aim to reduce the number of MetS components among those individuals with little epigenetic age acceleration may benefit the most from such modifications with regard to the aging of the epigenome, although additional studies are needed to verify this hypothesis.

We also observed that advanced epigenetic age acceleration was associated with MetS development at later stages in life, i.e., IEAA at year 20 was associated with MetS at year 30. This finding strengthens the hypothesis that biological age may be useful in assessing risk of metabolic health outcomes and adds to the growing body of evidence that accelerated epigenetic aging may predict future health events. Numerous studies have shown that epigenetic age can be used in predicting cancer mortality [18], cardiovascular mortality [20], and lung cancer [24]. These findings aid in elucidating the role of epigenetic aging on different health states, as well as demonstrate the potential utility of this biological marker for aging in risk prediction. We did not, however, observe significant associations with epigenetic age acceleration at year 15 and MetS status at year 25, possibly due to relatively younger epigenomes and an overall healthier population at these time points. Moreover, later time points may better capture both the cumulative aging effects on the epigenome and the greater amount of age-related metabolic disturbances, and as such, we would expect to see more associations with older aged individuals when the phenotype is more prevalent. With regard to the MetS severity score, more of the metabolic factors presented in this study are associated with IEAA than with EEAA. IEAA and EEAA are two measures of epigenetic age acceleration that are based on different sets of CpGs and capture different biological processes of aging. IEAA is proposed to reflect cell-intrinsic aging that appears unaffected by lifestyle factors, whereas EEAA is proposed to reflect age-related alterations in leukocyte composition and correlates with lifestyle characteristics [25]. Based on these inherent differences, it is not unexpected that different associations were observed between the two measures. Moreover, similar findings were previously observed in other study populations, i.e., MetS was more significantly associated with IEAA than EEAA [16]. Thus, our findings, in conjunction with previous findings, suggest that cell-intrinsic aging, rather than immune system aging, may contribute more to MetS and its related components.

The large sample size of this study enabled us to obtain stable estimates for the conducted analyses. Additionally, the longitudinal aspect of this study provided us the opportunity to examine the temporal relationship between EAA and MetS. However, this study is not without limitations. We found differences in sample characteristics between participants with and without methylation data at years 15 and 20. A possible explanation for the difference in sex is the manner in which participants were selected for methylation profiling. Given study participants were stratified by sex (1:1 ratio of females and males) for profiling, the methylation cohorts’ relatively even sex distributions would be expected, as would the difference from the full cohort (which had a greater proportion of females). However, as both subgroups selected for methylation processing remained well-balanced on almost all other characteristics, and because our analyses both control for and stratify on sex and race, we believe that these differences do not impact the present analysis. Future research in CARDIA using DNA methylation from the full study population may help clarify any lingering concern over differences in our study population. Our analysis of MetS cases was limited by the relative youth of our population; we were unable to evaluate the examined associations in older individuals (chronological age > 60 years) where MetS is more prevalent. Further studies are needed to evaluate the association between EAA and MetS among this age group. Although this served as a strength for examining early metabolic changes that may presage MetS, and thus allowed us to evaluate the predictive efficacy of epigenetic age acceleration, future research with greater longitudinal follow-up may be able to provide even more concrete validation of our findings. Similarly, while the CARDIA population is very diverse in its characteristics, the study design was limited to four geographic areas of the USA and to white and African-American populations. Additional validation of these findings in more diverse populations in race/ethnicity and geographic areas will allow their generalizability to the population as a whole. And lastly, several analyses were performed, raising a potential issue with multiple testing. The current study sought to evaluate the associations between different measures of EEA at different time points of MetS and inherently yielded multiple analyses. We did not formally correct for multiple testing as the analyses were primarily non-independent, and we wanted to avoid increasing the chance of false-negative results that may hinder future investigations [26]. The analyses presented are complements of each other and thus does not necessitate correction for multiple testing. Moreover, our analyses yielded consistent results, both at individual time points and during repeated measures, indicating the reliability of the findings and our conclusions are based on general patterns of associations that are consistent with previous findings.

## Conclusions

In conclusion, we identified significant associations between the MetS severity score and both IEAA and EEAA cross-sectionally, suggesting with a greater number of MetS components associated with more advanced epigenetic age acceleration. Prospectively, we identified significant associations between epigenetic age acceleration and incident MetS. These findings provide novel insight into the relationship between epigenetic aging and MetS by indicating metabolic risk factors may accelerate the biological aging process and epigenetic markers of aging may serve as a predictive tool in the development of metabolic disorders. Moreover, additional studies will aid in identifying causal relationships that may improve the predictive ability for MetS, such as Mendelian randomization. Epigenetic age acceleration has the potential to be adapted into an early detection biomarker of age-related chronic diseases, which may have a large impact on public health as the population ages. Moreover, given the movement towards precision medicine, the epigenome may serve as an additional source of information to summarize an individual’s overall risk to disease and subsequently may provide novel information for the development of disease prevention strategies.

## Methods

### Study sample

Participants from the CARDIA study were included in this analysis. Details regarding the study design, recruitment, and examinations have been described elsewhere [27]. Briefly, the CARDIA study was established to investigate the development and determinants of subclinical and clinical cardiovascular disease and related risk factors. A total of 5115 black and white study participants 18 to 30 years of age were recruited from 4 centers (Birmingham, AL; Chicago, IL; Minneapolis, MN; and Oakland, CA) at the study baseline from 1985 to 1986. Study participants were followed over time and received examinations at 2, 5, 7, 10, 15, 20, 25, and 30 years after the baseline visit. For the current study, we selected a subset of participants who had available whole blood at years 15 and 20. A total of 1200 participants, stratified by sex to ensure equal representation of this demographic characteristic, were randomly selected for methylation profiling. Study participants with methylation data at years 15 and 20 and those with complete clinical data relating to the MetS components at years 15, 20, 25, and 30 were included in this analysis. In total, 1024 and 923 study participants had available whole blood and complete MetS data at years 15 and 20, respectively. Additionally, among those with available whole blood at year 15 who were non-cases, 860 and 738 study participants had MetS data at years 25 and 30, respectively. For those with available whole blood at year 20 who were non-cases, 678 and 586 study participants had MetS data at years 25 and 30, respectively.

### Metabolic syndrome severity score and case definition

As per guidelines from the American Heart Association and the National Heart, Lung, and Blood Institute, we used the following five components and criteria to define MetS: abdominal obesity (waist circumference ≥ 88 cm for women and ≥ 102 cm for men), elevated triglyceride levels (≥ 150 mg/dL or drug treatment for elevated triglycerides), low HDL cholesterol levels (< 50 mg/dL for women or < 40 mg/dL for men or drug treatment for reduced HDL), elevated blood pressure (systolic blood pressure ≥ 130 mmHg or diastolic blood pressure ≥ 85 mmHg, or use of medication to treat hypertension), or elevated fasting blood glucose levels (≥ 100 mg/dL or current use of medication to treat hyperglycemia) [28]. In order to better account for blood glucose levels, participants who control diabetes with either diabetic medications or diet and pills only were classified as having elevated fasting glucose. The MetS severity score was calculated for each study participant as the summation of the number of MetS components, ranging from 0 to 5. Study participants with at least three components were defined as MetS cases and participants with 2 or less were defined as non-cases.

### DNA methylation profiling

Standard methods were used for DNA extraction and quality control (available upon request). DNA methylation levels were estimated at years 15 and 20 of CARDIA using the Illumina MethylationEPIC Beadchip (~ 850,000 sites). Samples that contained > 5% failed probes at detection *P* > 0.01, CpG sites that failed in > 5% of samples (i.e., call rate < 95%), and CpGs containing common genetic variants based on dbSNP were removed [29, 30]. The final analysis set included only CpG probes located on autosomal chromosomes, while CpGs on chromosomes X and Y were retained to examine concordance between biological sex and self-reported sex. Cytosine modification intensities were generated using the R package *minfi* [31].

### Epigenetic age and EAA calculation

We estimated epigenetic age using two methods available through Horvath’s online epigenetic age calculator (http://labs.genetics.ucla.edu/horvath/dnamage/). The first method, developed by Horvath, uses DNA methylation levels at 353 CpG sites [15]. Intrinsic epigenetic age acceleration (IEAA) was then estimated from the residuals from a linear regression model of epigenetic age regressed on chronological age and numerous blood immune cell counts. As such, IEAA is proposed to capture cell-intrinsic properties of the aging process, independent of blood cell composition. The second method, developed by Hannum, uses DNA methylation levels at 71 CpG sites [32]. Similar to IEAA, extrinsic epigenetic age acceleration (EEAA) was estimated by calculating the residuals from a linear regression model of epigenetic age regressed on chronological age, up-weighting age-related blood cell counts. EEAA is proposed to reflect immune system aging. These estimations were performed for study participants at years 15 and 20. Both IEAA and EEAA measured were calculated and reported to be consistent with a previous study investigating MetS and EAA, as well as to elucidate the biological mechanism of each EAA measure on MetS.

### Statistical analysis

We first performed cross-sectional analyses to examine associations between MetS severity score and IEAA and EEAA at years 15 and 20, to determine whether individuals with higher MetS severity scores (more MetS components) exhibited greater epigenetic age acceleration. We performed linear regression with epigenetic age acceleration (IEAA or EEAA) modeled as the outcome and the MetS severity score as the independent variable. Quantile regression was additionally performed to further assess the relationship between epigenetic age acceleration, as the outcome, and the MetS severity score, as the independent variable. This approach provides a more complete picture of covariate effects on the variables under investigation [33]. We also evaluated generalized estimating equations (GEEs) to further examine the associations between the MetS severity score and epigenetic age acceleration, a nuanced approach that previously has been used to evaluate the association of epigenetic age acceleration across time on outcomes of interest [34]. GEE captures and accounts for within-individual variation with repeated measurements of methylation data and represents a larger effective sample size, providing more stable parameter estimates for the associations under investigation. We then assessed whether epigenetic age acceleration at years 15 and 20 was associated with incident MetS status at years 25 and 30 using logistic regression, and further evaluated these associations to leverage repeated measures using GEEs. We further explored sex and race interactions with the exposure variable for each model. All models were adjusted for sex, race, center, education, smoking status, alcohol consumption, and physical activity and all statistical analyses performed using SAS 9.4.

## Supplementary information

**Additional file 1:** **Table S1.** Comparison of study sample characteristics between participants who underwent methylation profiling and participants who did not at examination years 15 and 20, adjusting for race and sex.

**Additional file 2:** **Figure S1.** Scatterplot matrix of chronological age, biological age, and MetS severity score at examination years 15 and 20. Scatterplot matrix displaying chronological age, epigenetic age as calculated by Horvath’s and Hannum’s method, and the MetS severity score at examination years 15 and 20.

**Additional file 3:** **Figure S2.** Estimated parameters by quantile with 95% confidence limits for the effect of the MetS severity score on EEAA. Quantile regression plots for the MetS severity score at years (A) 15 and (B) 20. The x axis represents the quantile scale and the y axis represents the effect of the MetS severity score on EEAA for a given quantile. Results are adjusted for sex, race, center, education, smoking status, alcohol consumption, and physical activity.

## Data Availability

The epigenetic datasets generated and analyzed are available from the corresponding author on reasonable request.

## Abbreviations

CARDIA: Coronary Artery Risk Development in Young Adults
EAA: Epigenetic age acceleration
EEAA: Extrinsic epigenetic age acceleration
GEE: Generalized estimating equations
HDL: High-density lipoprotein
IEAA: Intrinsic epigenetic age acceleration
MetS: Metabolic syndrome
OR: Odds ratio

## References

[1] Grundy SM, Cleeman JI, Daniels SR, Donato KA, Eckel RH, Franklin BA (2005). Diagnosis and management of the metabolic syndrome. Circulation..

[2] Wilson PW, D’Agostino RB, Parise H, Sullivan L, Meigs JB (2005). Metabolic syndrome as a precursor of cardiovascular disease and type 2 diabetes mellitus. Circulation..

[3] Bjorge T, Lukanova A, Jonsson H, Tretli S, Ulmer H, Manjer J (2010). Metabolic syndrome and breast cancer in the me-can (metabolic syndrome and cancer) project. Cancer Epidemiol Biomark Prev.

[4] Lindkvist B, Johansen D, Stocks T, Concin H, Bjorge T, Almquist M (2014). Metabolic risk factors for esophageal squamous cell carcinoma and adenocarcinoma: a prospective study of 580,000 subjects within the Me-Can project. BMC Cancer.

[5] Isomaa B, Almgren P, Tuomi T, Forsen B, Lahti K, Nissen M (2001). Cardiovascular morbidity and mortality associated with the metabolic syndrome. Diabetes Care.

[6] Wang J, Ruotsalainen S, Moilanen L, Lepisto P, Laakso M, Kuusisto J (2007). The metabolic syndrome predicts cardiovascular mortality: a 13-year follow-up study in elderly non-diabetic Finns. Eur Heart J.

[7] Saklayen MG (2018). The global epidemic of the metabolic syndrome. Curr Hypertens Rep.

[8] Moore JX, Chaudhary N, Akinyemiju T (2017). Metabolic syndrome prevalence by race/ethnicity and sex in the United States, National Health and Nutrition Examination Survey, 1988-2012. Prev Chronic Dis.

[9] Aguilar M, Bhuket T, Torres S, Liu B, Wong RJ (2015). Prevalence of the metabolic syndrome in the United States, 2003-2012. JAMA..

[10] Revesz D, Milaneschi Y, Verhoeven JE, Penninx BW (2014). Telomere length as a marker of cellular aging is associated with prevalence and progression of metabolic syndrome. J Clin Endocrinol Metab.

[11] Revesz D, Milaneschi Y, Verhoeven JE, Lin J, Penninx BW (2015). Longitudinal associations between metabolic syndrome components and telomere shortening. J Clin Endocrinol Metab.

[12] Harte AL, da Silva NF, Miller MA, Cappuccio FP, Kelly A, O'Hare JP (2012). Telomere length attrition, a marker of biological senescence, is inversely correlated with triglycerides and cholesterol in South Asian males with type 2 diabetes mellitus. Exp Diabetes Res.

[13] Zannolli R, Mohn A, Buoni S, Pietrobelli A, Messina M, Chiarelli F (2008). Telomere length and obesity. Acta Paediatr.

[14] Lee M, Martin H, Firpo MA, Demerath EW (2011). Inverse association between adiposity and telomere length: the Fels Longitudinal Study. Am J Hum Biol.

[15] Horvath S (2013). DNA methylation age of human tissues and cell types. Genome Biol.

[16] Quach A, Levine ME, Tanaka T, Lu AT, Chen BH, Ferrucci L (2017). Epigenetic clock analysis of diet, exercise, education, and lifestyle factors. Aging (Albany NY).

[17] Breitling LP, Saum KU, Perna L, Schottker B, Holleczek B, Brenner H (2016). Frailty is associated with the epigenetic clock but not with telomere length in a German cohort. Clin Epigenetics.

[18] Zheng Y, Joyce BT, Colicino E, Liu L, Zhang W, Dai Q (2016). Blood epigenetic age may predict cancer incidence and mortality. EBioMedicine..

[19] Marioni RE, Shah S, McRae AF, Ritchie SJ, Muniz-Terrera G, Harris SE (2015). The epigenetic clock is correlated with physical and cognitive fitness in the Lothian Birth Cohort 1936. Int J Epidemiol.

[20] Perna L, Zhang Y, Mons U, Holleczek B, Saum KU, Brenner H (2016). Epigenetic age acceleration predicts cancer, cardiovascular, and all-cause mortality in a German case cohort. Clin Epigenetics.

[21] Veronica G, Esther RR (2012). Aging, metabolic syndrome and the heart. Aging Dis.

[22] Bonomini F, Rodella LF, Rezzani R (2015). Metabolic syndrome, aging and involvement of oxidative stress. Aging Dis.

[23] Yara S, Lavoie JC, Levy E (2015). Oxidative stress and DNA methylation regulation in the metabolic syndrome. Epigenomics..

[24] Levine ME, Hosgood HD, Chen B, Absher D, Assimes T, Horvath S (2015). DNA methylation age of blood predicts future onset of lung cancer in the women’s health initiative. Aging (Albany NY).

[25] Lu AT, Xue L, Salfati EL, Chen BH, Ferrucci L, Levy D (2018). GWAS of epigenetic aging rates in blood reveals a critical role for TERT. Nat Commun.

[26] Rothman KJ (1990). No adjustments are needed for multiple comparisons. Epidemiology..

[27] Friedman GD, Cutter GR, Donahue RP, Hughes GH, Hulley SB, Jacobs DR (1988). CARDIA: study design, recruitment, and some characteristics of the examined subjects. J Clin Epidemiol.

[28] Grundy SM, Cleeman JI, Daniels SR, Donato KA, Eckel RH, Franklin BA (2005). Diagnosis and management of the metabolic syndrome: an American Heart Association/National Heart, Lung, and Blood Institute scientific statement. Circulation..

[29] Moen EL, Zhang X, Mu W, Delaney SM, Wing C, McQuade J (2013). Genome-wide variation of cytosine modifications between European and African populations and the implications for complex traits. Genetics..

[30] Zhang X, Mu W, Zhang W (2012). On the analysis of the Illumina 450k array data: probes ambiguously mapped to the human genome. Front Genet.

[31] Aryee MJ, Jaffe AE, Corrada-Bravo H, Ladd-Acosta C, Feinberg AP, Hansen KD (2014). Minfi: a flexible and comprehensive Bioconductor package for the analysis of Infinium DNA methylation microarrays. Bioinformatics..

[32] Hannum G, Guinney J, Zhao L, Zhang L, Hughes G, Sadda S (2013). Genome-wide methylation profiles reveal quantitative views of human aging rates. Mol Cell.

[33] Koenker R, Hallock KF (2001). Quantile regression. J Econ Perspect.

[34] Binder AM, Corvalan C, Mericq V, Pereira A, Santos JL, Horvath S (2018). Faster ticking rate of the epigenetic clock is associated with faster pubertal development in girls. Epigenetics..

