# Supplementary material for: Epigenetic age acceleration and metabolic syndrome in the coronary artery risk development in young adults study
Source: Clin Epigenetics. 2019 Nov 15;11:160. doi: 10.1186/s13148-019-0767-1 (PMC6858654; doi:10.1186/s13148-019-0767-1)
Supplement: Supplementary file 1 — Additional file 1: Table S1. Comparison of study sample characteristics between participants who underwent methylation profiling and participants who did not at examination years 15 and 20, adjusting for race and sex. [file 13148_2019_767_MOESM1_ESM.docx]

Additional file 1: Table S1. Comparison of study sample characteristics between participants who underwent methylation profiling and participants who did not at examination years 15 and 20, adjusting for race and sex

|  | Year 15 Cohort | | |  | Year 20 Cohort | | |
| --- | --- | --- | --- | --- | --- | --- | --- |
| Characteristic^a^ | Without  Methylation | With  Methylation | *P* |  | Without  Methylation | With  Methylation | *P* |
| N | 2,630 | 1,042 |  |  | 2,592 | 957 |  |
| Female, n (%) | 1517 (57.7) | 535 (51.3) | 0.0005 |  | 1,524 (58.8) | 490 (51.2) | 0.0001 |
| Race, n (%) |  |  |  |  |  |  |  |
| White | 1,324 (50.3) | 618 (59.3) |  |  | 1,334 (51.5) | 564 (58.9) |  |
| Black | 1,306 (49.7) | 424 (40.7) | 0.0001 |  | 1,258 (48.5) | 393 (41.1) | 0.0001 |
| Age, mean (SD), y | 40.1 (3.7) | 40.4 (3.5) | 0.16 |  | 45.1 (3.7) | 45.4 (3.5) | 0.19 |
| Epigenetic age, mean (SD), y^b^ | N/A | 45.4 (5.5) |  |  | N/A | 49.4 (6.1) |  |
| IEAA, mean (SD), y | N/A | 0 (4.6) |  |  | N/A | 0 (5.4) |  |
| EEAA, mean (SD), y | N/A | 0 (6.4) |  |  | N/A | 0 (5.4) |  |
| Education, mean (SD), y | 14.8 (2.5) | 15.1 (2.5) | 0.22 |  | 15.0 (2.6) | 15.1 (2.5) | 0.96 |
| Center, n (%) |  |  |  |  |  |  |  |
| Birmingham, AL | 603 (22.9) | 255 (24.5) |  |  | 597 (23.0) | 222 (23.2) |  |
| Chicago, IL | 591 (22.5) | 225 (21.6) |  |  | 585 (22.6) | 208 (21.7) |  |
| Minneapolis, MN | 715 (27.2) | 278 (26.7) |  |  | 667 (25.7) | 258 (27.0) |  |
| Oakland, CA | 721 (27.4) | 284 (27.3) | 0.78 |  | 743 (28.7) | 269 (28.1) | 0.87 |
| Smoking Status, n (%) |  |  |  |  |  |  |  |
| Never | 1,540 (58.7) | 653 (62.8) |  |  | 1,580 (61.5) | 570 (60.2) |  |
| Former | 484 (18.4) | 181 (17.4) |  |  | 491 (19.1) | 191 (20.2) |  |
| Current | 601 (22.9) | 206 (19.8) | 0.06 |  | 497 (19.4) | 186 (19.6) | 0.73 |
| Alcohol, mean (SD), mL/day | 10.5 (25.7) | 12.1 (22.6) | 0.32 |  | 10.0 (18.2) | 11.1 (18.4) | 0.1 |
| Physical Activity, mean (SD), total intensity score | 346.3 (287.1) | 350.0 (274.7) | 0.39 |  | 331.2 (273.3) | 348.6 (276.1) | 0.72 |
| BMI, mean (SD), kg/m^2^ | 28.8 (7.1) | 28.5 (6.2) | 0.9 |  | 29.5 (7.5) | 29.3 (6.5) | 0.9 |
| SBP, mean (SD), mmHg | 113.6 (15.4) | 112.3 (13.6) | 0.15 |  | 116.8 (15.4) | 116.4 (14.9) | 0.65 |
| DBP, mean (SD), mmHg | 74.7 (11.9) | 74.1 (10.8) | 0.27 |  | 73.3 (11.6) | 72.7 (11.2) | 0.48 |
| Total cholesterol, mean (SD), mg/dL | 184.0 (35.9) | 186.4 (35.5) | 0.19 |  | 184.7 (34.6) | 188.3 (36.1) | 0.016 |
| HDL cholesterol, mean (SD), mg/dL | 50.9 (14.7) | 50.1 (14.1) | 0.65 |  | 54.6 (16.6) | 53.2 (16.7) | 0.36 |
| Triglycerides, mean (SD), mg/dL | 103.2 (92.5) | 110.9 (93.4) | 0.28 |  | 105.9 (72.6) | 119.0 (95.4) | 0.019 |
| Glucose, mean (SD), mg/dL | 86.8 (21.9) | 86.3 (18.4) | 0.42 |  | 98.1 (27.4) | 97.9 (23.8) | 0.76 |
| Waist circumference, mean (SD), cm | 89.5 (16.0) | 89.5 (13.9) | 0.92 |  | 91.8 (15.9) | 92.2 (14.6) | 0.83 |
| Metabolic Syndrome |  |  |  |  |  |  |  |
| Prevalence, n (%) | 426 (16.8) | 164 (15.9) | 0.53 |  | 625 (24.7) | 253 (26.5) | 0.28 |
| Severity score, median (IQR) | 1 (0, 2) | 1 (0, 2) |  |  | 1 (0, 2) | 1 (0, 3) |  |
| 0 components, n (%) | 942 (37.0) | 366 (35.5) |  |  | 757 (30.0) | 278 (29.1) |  |
| 1 components, n (%) | 678 (26.7) | 307 (29.8) |  |  | 669 (26.4) | 237 (24.8) |  |
| 2 components, n (%) | 498 (19.6) | 195 (18.9) |  |  | 482 (19.0) | 188 (19.7) |  |
| 3 components, n (%) | 279 (11.0) | 108 (10.5) |  |  | 334 (13.2) | 146 (15.3) |  |
| 4 components, n (%) | 115 (4.5) | 49 (4.8) |  |  | 219 (8.7) | 78 (8.2) |  |
| 5 components, n (%) | 32 (1.3) | 7 (0.7) | 0.33 |  | 72 (2.8) | 29 (3.0) | 0.63 |

^a^ Study sample characteristics were measured at years 15 and 20 of CARDIA, respectively.

^b^ DNA methylation age as predicted by Horvath’s method.
