# Supplementary material for: Epigenetic age acceleration and metabolic syndrome in the coronary artery risk development in young adults study
Source: Clin Epigenetics. 2019 Nov 15;11:160. doi: 10.1186/s13148-019-0767-1 (PMC6858654; doi:10.1186/s13148-019-0767-1)
Supplement: Supplementary file 2 — Additional file 2: Figure S1. Scatterplot matrix of chronological age, biological age, and MetS severity score at examination years 15 and 20. Scatterplot matrix displaying chronological age, epigenetic age as calculated by Horvath’s and Hannum’s method, and the MetS severity score at examination years 15 and 20. [file 13148_2019_767_MOESM2_ESM.docx]

Additional file 2: Figure S1. Scatterplot matrix of chronological age, biological age, and MetS severity score at examination years 15 and 20.


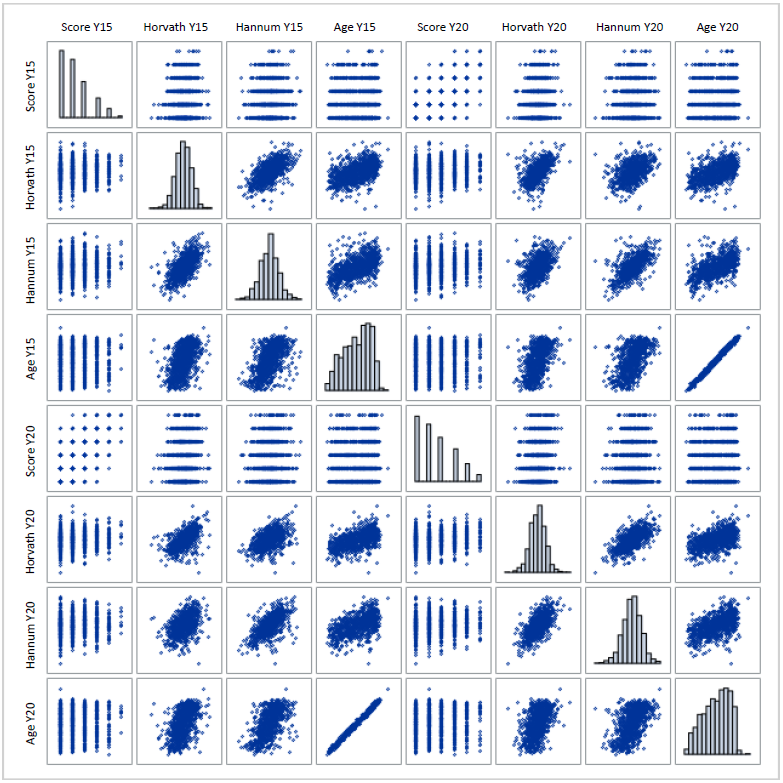


Scatterplot matrix displaying chronological age, epigenetic age as calculated by Horvath’s and Hannum’s method, and the MetS severity score at examination years 15 and 20.
