# Supplementary material for: Epigenetic age acceleration and metabolic syndrome in the coronary artery risk development in young adults study
Source: Clin Epigenetics. 2019 Nov 15;11:160. doi: 10.1186/s13148-019-0767-1 (PMC6858654; doi:10.1186/s13148-019-0767-1)
Supplement: Supplementary file 3 — Additional file 3: Figure S2. Estimated parameters by quantile with 95% confidence limits for the effect of the MetS severity score on EEAA. Quantile regression plots for the MetS severity score at years (A) 15 and (B) 20. The x axis represents the quantile scale and the y axis represents the effect of the MetS severity score on EEAA for a given quantile. Results are adjusted for sex, race, center, education, smoking status, alcohol consumption, and physical activity. [file 13148_2019_767_MOESM3_ESM.docx]

Additional file 3: Figure S2. Estimated parameters by quantile with 95% confidence limits for the effect of the MetS severity score on EEAA.

A. B.


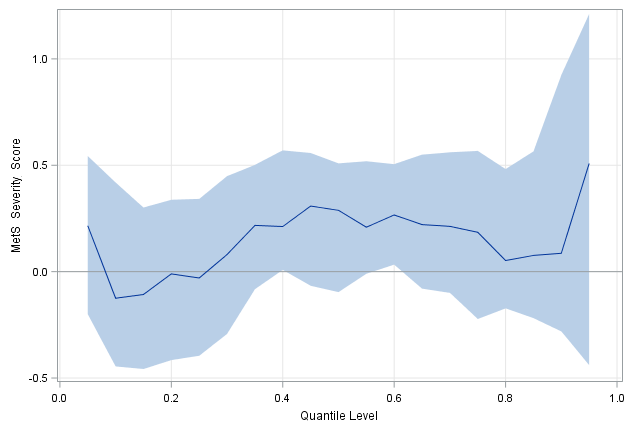

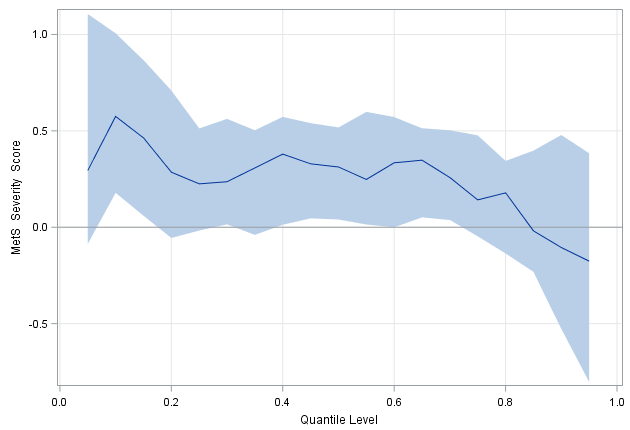


Quantile regression plots for the MetS severity score at years (A) 15 and (B) 20. The x axis represents the quantile scale and the y axis represents the effect of the MetS severity score on EEAA for a given quantile. Results are adjusted for sex, race, center, education, smoking status, alcohol consumption, and physical activity.
